# A novel small molecule inhibits STAT3 phosphorylation and DNA binding activity and exhibits potent growth suppressive activity in human cancer cells

**DOI:** 10.1186/1476-4598-9-217

**Published:** 2010-08-16

**Authors:** Li Lin, Stephanie Deangelis, Elizabeth Foust, James Fuchs, Chenglong Li, Pui-Kai Li, Eric B Schwartz, Gregory B Lesinski, Don Benson, Jiagao Lü, Dale Hoyt, Jiayuh Lin

**Affiliations:** 1 Department of Pediatrics, College of Medicine, The Ohio State University, Columbus, Ohio, 43205, USA; 2 Division of Cardiology, Department of Internal Medicine, Tongji Hospital, Tongji Medical College, Huazhong University of Science and Technology, Wuhan, 430030, China; 3 Division of Medicinal Chemistry and Pharmacognosy, College of Pharmacy, The Ohio State University, Columbus, Ohio, 43210, USA; 4 Department of Internal Medicine, The Ohio State University, Columbus, Ohio, 43210, USA; 5 Experimental Therapeutics Program, The Ohio State University Comprehensive Cancer Center, The Ohio State University, Columbus, Ohio, 43210, USA

## Abstract

**Background:**

Targeting Signal Transducer and Activator of Transcription 3 (STAT3) signaling is an attractive therapeutic approach for most types of human cancers with constitutively activated STAT3. A novel small molecular STAT3 inhibitor, FLLL32 was specifically designed from dietary agent, curcumin to inhibit constitutive STAT3 signaling in multiple myeloma, glioblastoma, liver cancer, and colorectal cancer cells.

**Results:**

FLLL32 was found to be a potent inhibitor of STAT3 phosphorylation, STAT3 DNA binding activity, and the expression of STAT3 downstream target genes *in vitro*, leading to the inhibition of cell proliferation as well as the induction of Caspase-3 and PARP cleavages in human multiple myeloma, glioblastoma, liver cancer, and colorectal cancer cell lines. However, FLLL32 exhibited little inhibition on some tyrosine kinases containing SH2 or both SH2 and SH3 domains, and other protein and lipid kinases using a kinase profile assay. FLLL32 was also more potent than four previously reported JAK2 and STAT3 inhibitors as well as curcumin to inhibit cell viability in these cancer cells. Furthermore, FLLL32 selectively inhibited the induction of STAT3 phosphorylation by Interleukin-6 but not STAT1 phosphorylation by IFN-γ.

**Conclusion:**

Our findings indicate that FLLL32 exhibits potent inhibitory activity to STAT3 and has potential for targeting multiple myeloma, glioblastoma, liver cancer, and colorectal cancer cells expressing constitutive STAT3 signaling.

## Introduction

The Signal Transducer and Activator of Transcription 3 (STAT3) protein is a member of the STAT family of transcription factors which are initially located in the cytoplasm in their inactive form. After stimulation by extracellular signals, such as cytokines, growth factors and hormones, Janus kinases (JAKs) are activated and then induce the phophorylatation of STAT3 at tyrosine residue 705 (Y705) [1]. Phosphorylated STAT3 proteins dimerize via their Src-homology 2 (SH2) domains, and translocate to the nucleus where they regulate the expression of numerous critical genes involved in cell cycle progression, proliferation, migration and invasion, and survival [1]. However, the constitutive activation of STAT3 is frequently detected in clinical samples from a wide range of human carcinoma and established human cancer cell lines, such as multiple myeloma, glioblastoma, colorectal and hepatocellular carcinoma [1-5]. Importantly, elevated levels of STAT3 phosphorylation were correlated with the tumor invasion, metastasis, and worse prognosis in colorectal, hepatocellular and other carcinoma [2-5].

Blocking constitutive STAT3 signaling in carcinoma cells by STAT3 antisense oligonucleotides, STAT3 small interfering RNAs (siRNAs), or stable transfection of dominant-negative STAT3 [5] can inhibit cancer cells growth, invasion and metastasis, and induce apoptosis. Furthermore, inhibition of constitutive STAT3 signaling by the JAK2 inhibitor, AG490 [6] suppressed the growth, and decreased the invasion of human hepatocellular carcinoma cells, and also induced apoptosis in multiple myeloma cells [7]. These findings suggest that constitutive STAT3 signaling is crucial to the survival, invasion, and growth of human carcinoma cells. Targeting the STAT3 pathway directly should be a promising and novel form of treatment for these human cancers. A few non-peptide STAT3 SH2 inhibitors were recently developed to inhibit STAT3 dimerization, including Stattic [8], STA-21 [9], and S3I-201 [10]. Several new inhibitors of JAK2, the upstream kinase of STAT3, such as AG490 [6], WP1066 [11] have also been reported.

We have recently developed a series of novel curcumin-derived small molecule inhibitors of the JAK2/STAT3 pathway. Curcumin is the primary bioactive compound isolated from turmeric, the dietary spice made from the rhizome of *Curcuma longa*. Curcumin is known to inhibit several targets closely associated with cancer cell proliferation, in particular JAK2/STAT3 pathway [12,13]. Because of its poor bioavailability and potency, curcumin has somewhat limited potential as an anti-cancer drug. However, we utilized curcumin as a lead compound to design new small molecule STAT3 inhibitors. One compound identified by our group, named as FLLL32, has been shown to selectively inhibit STAT3 phosphorylation, STAT3 DNA binding activities, cell viability, and induce apoptosis in multiple myeloma, glioblastoma, colorectal and hepatocellular carcinoma cancer cells with constitutively activated STAT3 signaling.

## Results

### FLLL32, a curcumin analog that is specifically designed to target STAT3

**Figure 1:**
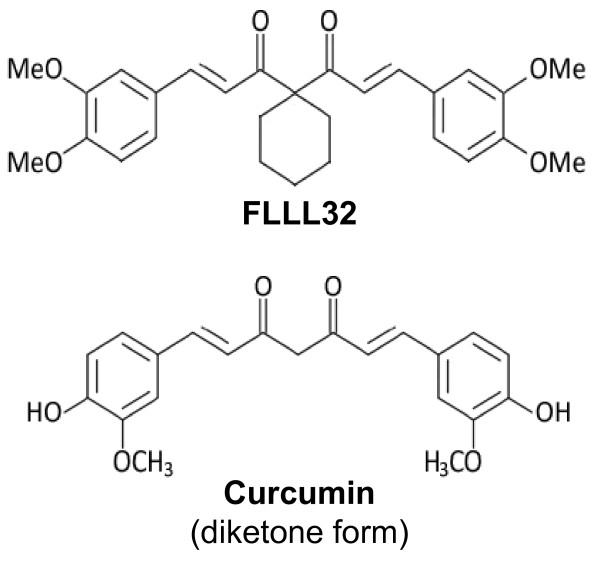
**The structures of FLLL32 and curcumin**. The chemical modifications made to FLLL32 prevent enolization and are proposed to confer greater stability and better access to critical domains in STAT3.

Computer models with molecular docking showed that only the keto form of curcumin binds to the STAT3 SH2 dimerization site (Table 1). However, curcumin exists almost entirely in the enol form in solution. FLLL32 is a diketone analogue of curcumin (Figure 1). FLLL32 was designed to lock its derivatives exclusively into the diketo form via substituting the two hydrogens on the middle carbon with spiro-cyloalkyl rings. Molecular docking showed that FLLL32 has better binding potencies to the STAT3 SH2 binding site (FLLL32 is 25-fold stronger in STAT3 SH2 binding) than the keto tautomer of curcumin (Table 1).

**Table 1:** Docking energies of curcumin and FLLL32 to STAT3

	Docking free energy (kcal/mol) to STAT3
Curcumin	-8.1(keto)
	
	no binding (enol)

FLLL32	-8.5

Curcumin has both enol and keto form. FLLL32 had better binding potency than curcumin.

### The STAT3 inhibitor, FLLL32 down-regulated STAT3 phosphorylation in cancer cells

**Figure 2:**
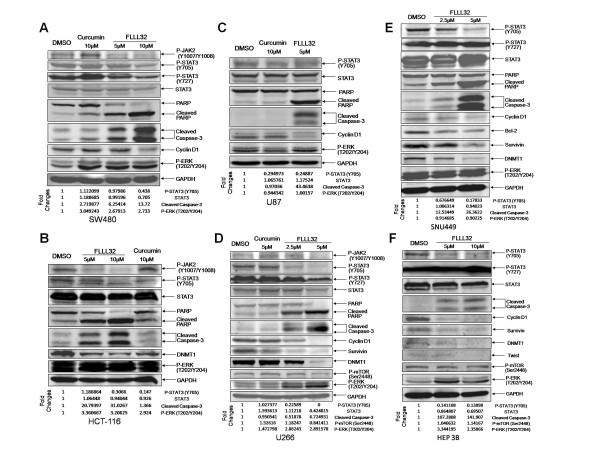
**FLLL32 inhibited STAT3 phosphorylation, and induced apoptosis in (A) SW480, (B) HCT-116 colorectal, (C) U87 glioblastoma, (D) U266 multiple myeloma, and (E) SNU449, (F) HEP3B liver cancer cells**. Cells were treated with FLLL32 (2.5-10 μM) or curcumin (5-10 μM) of for 24 hours. Decreases in P-STAT3 by FLLL32 were associated with decreased STAT3 downstream target genes and increased cleavage of caspase-3 and PARP. The phosphorylation of ERK1/2 and/or mTOR was not obviously reduced.

We first examined whether FLLL32 inhibits STAT3 phosphorylation at Tyrosine residue 705 (Y705). Phosphorylation of STAT3 at residue Y705 plays an important role in its activity and nuclear translocation. We detected the effects of FLLL32 on STAT3 phosphorylation by Western blots with a phospho-Y705-specific STAT3 antibody in a panel of glioblastoma, multiple myeloma, colorectal and liver cancer cell lines known to express high endogenous levels of constitutively activated STAT3. We found FLLL32 effectively decreased the levels of phosphorylated STAT3 (P-STAT3, Y705) in SW480 (Figure 2A) and HCT116 (Figure 2B) colorectal cancer cells and curcumin is not as potent as FLLL32. STAT3 is phosphorylated at tyrosine residue (Y705) and activated by upstream kinases such as Janus kinase 2 (JAK2) [14,15]. So we examined the phosphorylation of JAK2 (Y1007/1008) in these two colon cancer cell lines. We found that FLLL32 also inhibits JAK2 phosphorylation in both cell lines. FLLL32 with higher concentration (10 μM) also inhibited the phosphorylation of STAT3 at residue Ser727 in SW480 cancer cell line but in HCT116 cancer cell line, the phosphorylation of STAT3 (Ser 727) could not be detected (Figure 2A, 2B). The phosphorylation ERK1/2 was not inhibited by FLLL32 in both colon cancer cell lines (Figure 2A, B).

We next examined the effects of FLLL32 in U87 and U251 glioblastoma cells (Figure 2C and Additional File 1A). FLLL32 with higher concentration (10 μM) inhibited the phosphorylation of STAT3 at residue Ser727 in U251 glioblastoam cell line (Additional File 1A), but in U87 glioblastoama cell line the STAT3 Ser 727 phosphorylation could not be detected (Figure 2C). The phosphorylation ERK1/2 was not reduced by FLLL32 (Figure 2C and Additional File 1A). FLLL32 was also more potent than curcumin to inhibit STAT3 Y705 and JAK2 phosphorylation in U266 (Figure 2D) and ARH-77 (Additional File 1B) multiple myeloma cell lines. Higher concentration (5 μM) of FLLL32 also slightly inhibited the phosphorylation of STAT3 at residue Ser727 in both multiple myeloma cell lines.

The effects of STAT3 phosphorylation in liver cancer cells were also examined. FLLL32 inhibit STAT3 Y705 phosphorylation in SNU449 (Figure 2E), HEP3B (Figure 2F), SNU387 (Additional File 1C), and SNU398 (Additional File 1D) liver cancer cells. However, the phosphorylation of ERK1/2 was not reduced except in SNU387 cells. The phosphorylation of mTOR was also not reduced in HEP3B and SNU398 cells. FLLL32 has little effect in inhibiting STAT3 S727 phosphorylation in SNU449, HEP3B, SNU398 and liver cancer cells lines (Figure 2E, F, Additional File 1D). We were not able to detect JAK2 phosphorylation in these liver cancer cell lines and in SNU387 cell line, the phosphorylation of STAT3 (Ser727) could not be detected.

### FLLL32 inhibits the expression of the STAT3 downstream targets and induced apoptosis in cancer cells

FLLL32 was also found to down-regulate the expression of STAT3 downstream targets that are involved in cell proliferation, survival, and other functions. Not all of the cancer cell lines expressed the same STAT3 downstream targets but cyclin D1, Bcl-2, survivin, DNMT1 and TWIST1 were among the most common STAT3 downstream targets expressed and were inhibited by the STAT3 inhibitor, FLLL32 (Figure 2 and Additional File 1).

**Figure 3:**
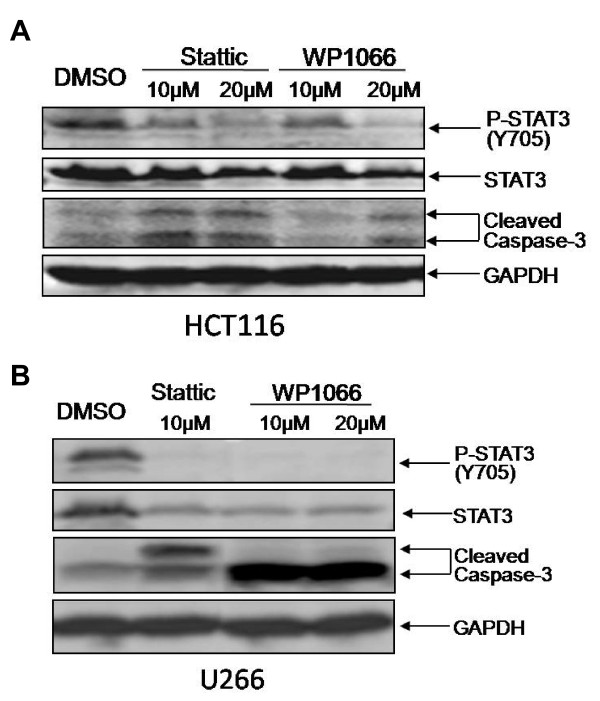
**Stattic (reported STAT3 inhibitor) and WP1066 (reported JAK2 inhibitor) were able to inhibit STAT3 phosphorylation and induce apoptosis indicated by increased cleaveage of capase-3 in (A) HCT116 colon cancer and (B) U266 multiple myeloma cells**.

With the decreases of STAT3 phosphorylation and STAT3 downstream targets, the induction of apoptosis by FLLL32 was as evidenced by cleaved poly-ADP ribose polymerase (PARP) PARP and caspase-3 in these human cancer cell lines (Figure 2 and Additional File 1). FLLL32 is also more potent than curcumin to induce apoptosis in these cancer cells. We also tested a previously reported STAT3 inhibitor Stattic [11] and a previously reported JAK2 inhibitor WP1066 [11] as positive controls to detect their effects on apoptosis. Stattic and WP1066 were also found to inhibit STAT3 phosphorylation and induce apoptosis indicated by the cleaveage of capase-3 in HCT116 colon cancer cells (Figure 3A) and U266 multiple myeloma cells (Figure 3B).

### FLLL32 inhibited STAT3 phosphorylation induced by IL-6 but not STAT1 phosphorylation induced by IFN-γ

**Figure 4:**
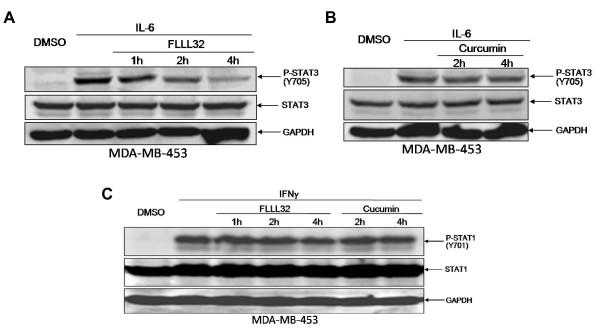
**Pre-treatment of cells with FLLL32 effectively inhibited the stimulation of STAT3 phosphorylation induced by IL-6, but had little impact on the phosphorylation of STAT1 induced by IFN-γ**.

Some of the cancer cells or cell lines employed in these studies do not express constitutively phosphorylated STAT3, such as the MDA-MB-453 breast cancer cell line. IL-6 is a cytokine which can induce the phosphorylation of STAT3 (Y705) [16-18]. We hypothesized that FLLL32 would be potent enough to inhibit IL-6 induced STAT3 phosphorylation. We found that pretreatment with FLLL32 but not curcumin (20 μM) was able to inhibit the induction of STAT3 phosphorylation by IL-6 in MDA-MB-453 breast cancer cells, and the effect of FLLL32 was more potent than curcumin (Figure 4A, B). However, pre-treatment of cells with FLLL32 had no impact on the phosphorylation of STAT1 induced by IFN-γ (Figure 4C). These results indicate the selectivity of FLLL32 on STAT3 but not STAT1.

### FLLL32 inhibited STAT3 DNA binding activity

**Figure 5:**
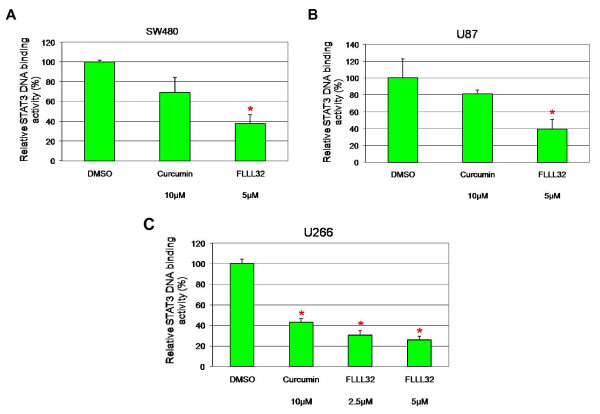
FLLL32 inhibited STAT3 DNA binding activity in (A) SW480 colorectal cancer cells, (B) U87 glioblastoma cells, and (C) U266 multiple myeloma cells (* P < 0.05).

After activation by phosphorylation at residue Y705, STAT3 dimerizes and translocates to the nucleus and induces the expression of downstream genes by binding specific DNA-response elements. We next examined the effect of FLLL32 on STAT3 DNA binding activity in U87 glioblastoma, U266 multiple myeloma and SW480 colorectal cancer cells. After 24 hours of treatment with FLLL32, the levels of STAT3 DNA-binding activity were decreased significantly in SW480, U87, and U266 cells (Figure 5A-C), and similarly the inhibitory effect of FLLL32 is more potent than curcumin (Figure 5A-C).

### Effects of FLLL32 on human protein and lipid kinases

We further examined whether FLLL32 inhibits other human kinase activity using a kinase profile assay. FLLL32 exhibited almost no inhibition (IC50 are greater than 100 μM) on tyrosine kinases containing SH2 or both SH2 and SH3 domains, such as JAK3, Lck, Syk, ZAP-70, TYK2, Abl-1, BTK, Lyn and Yes (Table 2). FLLL32 also exhibited little inhibition (IC50 are as high as 57.33 μM to 100 μM) on other protein kinases such as AKT1, CDK4/Cyclin D1, FAK, JNK1-α, mTOR, PI3K (P110α/85α, P110β/85α), PKA, PKCα, PKCγ (Table 2). As one of the positive controls, a known PI3K inhibitor, LY294002, the IC50 is 0.7853 μM. Several protein kinases (AKT, FAK and PKA) that were known to be inhibited by curcumin [19] were not inhibited by FLLL32 (Table 2). These results also support the specificity of FLLL32 to inhibit STAT3.

**Table 2:** The effect of FLLL32 on human protein and lipid kinases using a kinase profile assay.

	Protein Kinases	IC50 (μM)
Tyrosine kinases contain SH2 Domain	JAK3	>100
	
	Lck	>100
	
	Syk	>100
	
	ZAP-70	>100
	
	TYK2	>100

Tyrosine kinases containSH2 and SH3 Domains	Abl-1	>100
	
	BTK	>100
	
	Lyn	>100
	
	Yes	>100

Other human protein or lipid kinases	AKT1	>100
	
	CDK4/CyclinD1	>100
	
	FAK	>100
	
	JNK1-α1	>100
	
	MEK1	>100
	
	mTOR	>100
	
	PI3K (P110α/85α)	63.23
	
	PI3K (P110β/85α)	57.33
	
	PKA	>100
	
	PKCα	>100
	
	PKC-γ	>100

### The inhibitory efficacy of FLLL32 compared to other JAK2 and STAT3 inhibitors

Finally, the growth inhibitory activities of FLL32 were compared with those previously reported inhibitors in a panel of colorectal, glioblastoma, multiple myeloma and liver cancer cells lines. MTT assays were used to generate dose-response curves and evaluate cell viability following 72 hours of treatment with different concentrations of JAK2/STAT3 inhibitors, including FLLL32, WP1066, AG490, Stattic, S3I-201, and curcumin. The IC_50 _values of each compound in each cell line were calculated and listed in Table 3. In our testing, FLLL32 was more potent than other compounds in the growth suppression of each cell lines tested.

**Table 3:** IC_50 _values (μM) of FLLL32, curcumin, and other JAK2/STAT3 or STAT3 SH2 inhibitors in human colorectal cancer cells (C), glioblastoma cells (G), multiple myeloma (MM) and liver (L) cancer cells.

	FLLL32	WP1066	Stattic	S3I-201	AG490	Curcumin
SW480 (C)	0.38	1.98	0.43	>100	86.8	10.26

HCT-116 (C)	0.32	1.51	0.96	>100	50.5	10.91

U87 (G)	0.19	5.78	0.73	55.10	>100	6.91

U251 (G)	0.26	5.04	0.84	97.30	50.70	7.10

U266 (MM)	0.48	1.38	0.99	8.87	9.50	4.76

ARH77 (MM)	2.34	3.43	2.57	35.25	28.20	10.13

SNU-449 (L)	2.04	3.85	2.50	>100	>100	9.88

SNU-398 (L)	0.65	4.88	3.23	16.20	24.30	5.37

Hep3B (L)	2.31	10.20	17.78	>100	>100	68.12

SNU-387 (L)	2.89	5.17	4.30	>100	>100	25.54

### FLLL32 suppresses tumor growth *in vivo*

To determine the effect of FLLL32 to suppress tumor growth, mouse xenograft experiments were then performed to in an *in vivo *system. Two groups of 16 NON/SCID mice were obtained for tumor xenografts with the MDA-MB-231 breast cancer cell line. FLLL32 also could inhibit STAT3 phosphorylation and induce apoptosis in MDA-MB-231 breast cancer cells (Additional File 2A). After seeding and allowing the tumors to develop for 7 days, seven mice from each group were given daily intraperitoneal doses of 50 mg/kg FLLL32 whereas the other nine were given DMSO vehicle to serve as a control. The administration of FLLL32 resulted in significantly reduced tumor burdens in the MDA-MB-231 xenografts in mice compared to their DMSO-treated mice (Additional File 2B). These results indicated that FLLL32 not only potent in suppressing cancer cell growth *in vitro *but also potent in suppressing tumor grow in mice *in vivo*.

## Discussion

Colorectal cancer is the third most common form of cancer and the second most common cause of cancer-related death in the United States. Despite advances in the treatment of colorectal cancer, the five-year survival rate has only increased to 65%. Hence, novel therapeutic approaches of more effective treatments are much needed for colorectal cancer. The constitutive activation of STAT3 is frequently detected in primary human colorectal carcinoma cells and established human colorectal cancer cell lines [2,3,20,21] and elevated levels of STAT3 phosphorylation have been correlated with tumor invasion, nodal metastasis, and staging (P < 0.05) [3,20]. Additionally, constitutive STAT3 activation in colorectal cancer cells is associated with invasion, survival, and growth of colorectal cancer cells and the colorectal tumor model in mice *in vivo *[2,21-23]. These reports indicate that STAT3 is one of the major oncogenic pathways activated in colorectal cancer and can serve as a promising therapeutic target for colorectal carcinoma. Our data in this report demonstrated that, FLLL32, a novel STAT3 inhibitor, efficiently inhibited STAT3 phosphorylation, STAT3 DNA binding activity, which resulted the induction of apoptosis in human colorectal cancer cell lines.

The Signal Transducer and Activator of Transcription 3 (STAT3) signaling pathway has been implicated in the proliferation, chemoresistance, and survival of multiple myeloma cells [4,24]. Multiple myeloma is the second most common hematologic malignancy and will account for over 20,000 new diagnoses in 2009 in the United States. The incidence of the disease is rising and currently over 80,000 patients are living with multiple myeloma in the United States. Despite the advent of novel agents including lenalidomide and bortezomib, however, the disease remains incurable and new therapies are desperately needed. Our results presented in here also demonstrated that FLLL32 could efficiently inhibit STAT3 phosphorylation, STAT3 DNA binding activity, and induced of apoptosis in human multiple myeloma cell lines indicating that FLLL32 may be a potent therapeutic agent for this type of cancer with STAT3 is constitutively activated.

The third type of cancer we tested with FLLL32 is glioblastoma. Glioblastoma is the most common and aggressive of the primary brain tumors and 10,000 cases of glioblastoma are diagnosed in the United States each year. Glioblastoma continues to have very poor prognosis despite advances in chemotherapy and radiation therapy [25,26]. Many clinical cases of glioblastoma and glioblastoma cell lines express constitutively activated STAT3 [27,28]. Overexpression of IL-6, an upstream regulator of STAT3 [29] is also detected in glioblastoma and is a marker of malignancy [30,31]. The persistent activation of STAT3 is in part, also attributable to an autocrine action of IL-6 in the glioblastoma cells [32]. However, STAT3 was reported to play a pro-oncogenic or tumor-suppressive role depending on the the genetic background of the tumor [33]. Our results showed that FLLL32 was a potent inhibitor in inhibiting STAT3 phosphorylation and STAT3 DNA binding activity in human glioblastoma cell lines. Human glioblastoma cells were induced to apoptosis by the inhibition of STAT3 with FLLL32.

Furthermore, the inhibitory efficacy of FLLL32 in liver cancer cells was examined. Liver cancer or hepatocellular carcinoma is one of the most serious of cancers. According to the American Cancer Society, the five-year relative survival rates are currently at 11% for all stages, 7.7% for regional metastasis, and 2.9% for distant metastasis. Hence, there is an urgent need to develop more effective treatments for liver cancer. Patients with any stage of liver cancer may appropriately be considered candidates for clinical trials using new inhibitors because of the poor response to chemotherapy as conventionally used. The constitutive activation of STAT3 is frequently detected in clinical incidences of liver cancer and in more than 50% of human liver cancer cell lines but not in normal or non-transformed human cells [5,34,35]. The constitutive activation of STAT3 in liver cancer is frequently due to the aberrant methylation and silencing of Suppressor of Cytokine signaling-1 (SOCS-1) and -3 (SOCS-3) [34,35]. Constitutive STAT3 signaling contributes to liver cancer progression by promoting angiogenesis, survival, metastasis, and growth of liver cancer cells [5,34,35]. Again, our data demonstrated that FLLL32 could efficiently inhibit STAT3 phosphorylation and induced apoptosis in four independent human liver cancer cell lines. These results indicate that FLLL32 also has potential as a therapeutic agent for liver cancer cells expressing persistently activated STAT3.

In addition, FLLL32 also potent to inhibit STAT3 phosphorylation and induce apoptosis in MDA-MB-231 breast cancer cells. The potency of FLLL32 was further confirmed in MDA-MB-231 breast cancer xenografts in mouse model *in vivo*. Therefore, FLLL32 is not only potent in cancer cells in vitro but also in tumor cells in animal model in vivo and may have future potential to target tumor cells that express persistently activated STAT3 in cancer patients.

Curcumin has been demonstrated as a dietary agent that can inhibit STAT3[16,17]. FLLL32 was designed as a new analog which specifically targets STAT3 with higher binding potency and selectivity. Our data demonstrated that FLLL32 was more potent than curcumin to inhibit STAT3 phosphorylation and STAT3 DNA binding activity, downregulate STAT3 target genes, and induce cancer cells apoptosis. However, the phosphorylation of mTOR and ERK was not obviously reduced by FLLL32. FLLL32 also has little effect on STAT1 phosphorylation stimulated with IFN-γ. In addition, FLLL32 exhibited little inhibition on some of the tyrosine kinases containing SH2 or both SH2 and SH3 domains, and other protein kinases by using kinase profile assay. These results further support the specificity of FLLL32 to inhibit STAT3.

After activated by some cell surface cytokines, such as IL-6, IFN-γ, JAK2 phosphorylates and activates cytoplasmic STAT3 protein to an active dimer, which translocates to the nucleus and induce the transcription of specific target genes [14,15]. We found that FLLL32 inhibited P-JAK2 (Y1007/1008) in some of the cancer cell lines, which may explain the inhibition of the STAT3 phosphorylation in those cancer cell lines. Several new inhibitors of JAK2/STAT3 pathway were recently reported, such as Stattic [8], STA-21 [9], S3I-201 [10], AG490 [6], WP1066 [11]. Here, Stattic and WP1066 were used as positive control to detect their effects on apoptosis in HCT116 colon cancer and U266 multiple myeloma cells, which conformed the JAK2/STAT3 pathway may be an important target to induce the apoptosis of cancer cells. Furthermore, FLLL32 was found to be potent than other reported JAK2/STAT3 inhibitors, including FLLL32, WP1066, AG490, Stattic, S3I-201, and curcumin in our cancer cell lines.

## Conculsions

Our results have demonstrated that FLLL32 is an effective STAT3 inhibitor to inhibit STAT3 phophorlation, STAT3 DNA binding activity, STAT3 downstream target gene expression and induce apoptosis in human cancer cells from four independent cancer types such as multiple myeloma, glioblastoma, colorectal and liver cancers. FLLL32 was more potent than curcumin and other reported JAK2/STAT3 inhibitors in the inhibition of cancer cell viability in our comparisons. Our results suggest that FLLL32 is a potent therapeutic agent for multiple types of cancer cells expressing constitutive STAT3 signaling including multiple myeloma, glioblastoma, colorectal and liver cancer cells.

## Methods

### Cell Culture

Human colonrectal cancer cell lines (SW480, HCT116), glioblastoma cell line (U87, U251), human hepatic cancer cell lines (SNU-449, SNU-398, HEP3B and SNU387), human multiple myeloma cell line (U266 and ARH-77) and human breast cancer cell lines (MDA-MB-453, MDA-MB-231) were purchased from the American Type Culture Collection (Manassass, VA). These cancer cell lines were cultured in DMEM or RPMI-1640 supplemented with 10% fetal bovine serum.

### Inhibitors

FLLL32, a curcumin-derived STAT3 inhibitor, and WP1066 [11], a Janus-like kinase 2 (JAK2) inhibitor, were synthesized in Dr. Pui-Kai Li's laboratory (College of Pharmacy, The Ohio State University). STAT3 SH2 inhibitors Stattic [8] and S3I-201 [10], JAK2 inhibitor AG490 [6] was purchased from Calbiochem (San Diego, CA). Curcumin was purchased from Sigma-Aldrich Chemical Co. (Milwaukee, WI).

### Western blot analysis

FLLL32 and curcumin were dissolved in DMSO. Cancer cells were treated with the listed concentrations of these agents or DMSO for 24 hours, then lysed in cold RIPA lysis buffer containing protease inhibitors and subjected to SDS-PAGE. The primary antibodies were purchased from Cell Signaling Technologies (Danvers, MA, USA), including phospho-specific STAT3 (Tyrosine 705), phospho-specific STAT3 (Serine 727), phospho-specific JAK2 (Tyrosine1007/1008), phospho-specific STAT1 (Tyrosine 701), phospho-specific ERK1/2 (Threonine 202/Tyrosine 204), phospho-specific mTOR (Serine 2448), cleaved Poly (ADP-ribose) polymerase (PARP), cleaved caspase-3, cyclin D, Bcl-2, survivin, TWIST1 and GAPDH. DNMT1 primary antibodies were purchased from abcam Inc (Washington, DC). Membranes were analyzed with enhanced chemiluminescence Plus reagents (GE Healthcare) and scanned with a Storm PhosphorImager (Amersham Pharmacia Biotech Inc.).

### Kinase activity assay

The possible effects of FLLL32 on ten purified human protein kinases were performed at Reaction Biology Corp. (Malvern, PA) using Kinase profiler assay. The IC50 inhibitory values of FLLL32 on the kinase activity were determined using 10 different concentrations of FLLL32 with 100 μM as the highest concentration.

### IL-6 induction of STAT3 phosphorylation

MDA-MB-453 breast cancer cells were seeded and serum starved overnight. The cells were then left untreated or were treated with FLLL32 (20 μM), curcumin (20 μM) or DMSO for indicated hours. After stimulation with IL-6 (50 ng/mL, Sigma) or IFN-γ (50 ng/mL, Sigma) for 30 min, the cells were harvested and analyzed by western blot.

### STAT3 DNA-binding assays

After treatment with FLLL32, curcumin, or DMSO for 24 hours, the nuclear extract kit (Clontech Inc., Mountain View, CA) was used to prepare cell nuclear extracts following the manufacturer's protocol. Nuclear extracts were analyzed for STAT3 DNA binding activity using the TransFactor Universal STAT3-specific kits (Clontech Inc., Mountain View, CA) with an ELISA-based method.

### MTT cell viability assay

Cells were seeded in 96-well plates (3,000 cells/well) in triplicate, and treated with FLLL32 (0.1-10 μmol/L), curcumin (0.5-100 μmol/L), WP1066 (0.5-30 μmol/L), Stattic (0.5-30 μmol/L), S3I-201 (1-100 μmol/L), or AG490 (1-100 μmol/L) for 72 hours. Twenty-five μl of 3-(4,5-Dimethylthiazolyl)-2,5-diphenyltetrazolium bromide (MTT, Sigma) was added to each sample and incubated for 3.5 hours. After this, 100 μl of N, N-dimethylformamide (Sigma) solubilization solution was added to each well. The absorbance at 595 nm was read the following day. Half-Maximal inhibitory concentrations (IC_50_) were determined using Sigma Plot 9.0 software (Systat Software Inc., San Jose, CA).

### Mouse xenografts

All animal studies were conducted in accordance with the standard procedures approved by IACUC at the Research Institute at nationwide children's hospital. MDA-MB-231 breast cancer cells (5 × 10^6 ^in Matrigel) were implanted subcutaneously into the flank region of 4-6-week-old female NOD/SCID mice. After tumors developed (7 days), the mice were randomized into two groups and treated with 50 mg/kg FLLL32 (7 mice) or DMSO (9 mice) intraperitoneally daily for 18 days. Tumor growth was determined by measuring the major (L) and minor (W) diameter with a caliper. The tumor volume was calculated according to the formula: Tumor volume = 0.5236 × L × W^2^.

## Abbreviations

DMEM: Dulbecco's Modified Eagle Medium; DMSO: Dimethyl sulfoxide; GAPDH: Glyceraldehyde-3-phosphate dehydrogenase; MTT: 3-(4,5-Dimethylthiazolyl)-2,5-diphenyltetrazolium bromide; PARP: Poly ADP ribose polymerase; STAT: Signal Transducer and Activator of Transcription; Tyr705: Tyrosine 705.

## Competing interests

The authors declare that they have no competing interests.

## Authors' contributions

LL participated in experiment designs, coordinated the experiments, carried out western blot analysis, DNA-binding assay, contributed to the analysis and interpretation of data, and drafted the manuscript. SD and EF carried out the MTT assay. CL designed FLLL32. JF and PKL participated in the synthesis of FLLL32 and WP1066. JGL provided extensive support to LL to complete the experiments. GBL, DB, and DH contributed to the discussion of the experiments and research ideas. JL conceived the ideas, coordinated the experiments and supervised on the data analyses, interpretation and the manuscript draft. All authors read and approved the final manuscript.

## Supplementary Material

Additional file 1 **FLLL32 inhibited STAT3 phosphorylation in (A) U251 glioblastoma cells lines. (B) ARH-77 multiple myeloma, (C) SNU387 and (D) SNU398 liver cancer cells**. FLLL32 also inhibited the expression of Cyclin D1 and/or Bcl-2, DNMT1 and increased the cleavages of caspase-3 and PARP. Click here for file

Additional file 2 **The effect of FLLL32 on tumor growth *in vivo***. (A) FLLL32 inhibited STAT3 phosphorylation and induced apoptosis in MDA-MB-231 breast cancer cells lines; (B) FLLL32 suppressed the growth of MDA-MB-231 xenograft tumors in NOD/SCID mice. Click here for file
