# Supplementary figures and images for: A novel small molecule inhibits STAT3 phosphorylation and DNA binding activity and exhibits potent growth suppressive activity in human cancer cells
Source: Mol Cancer. 2010 Aug 16;9:217. doi: 10.1186/1476-4598-9-217 (PMC2936338; doi:10.1186/1476-4598-9-217)

## Slide 1
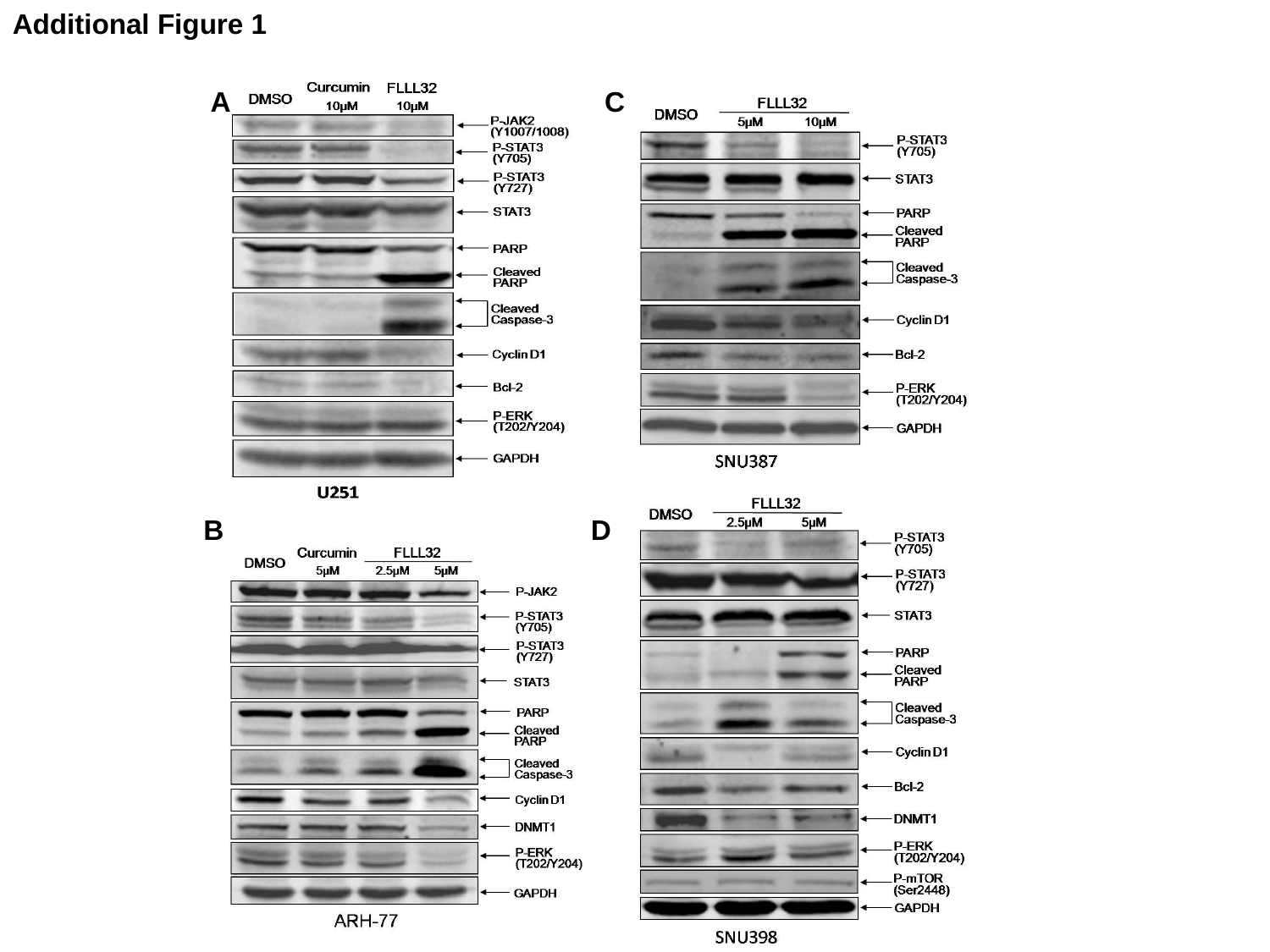

Additional Figure 1
A C
B D

Supplement: Additional file 1 — FLLL32 inhibited STAT3 phosphorylation in (A) U251 glioblastoma cells lines. (B) ARH-77 multiple myeloma, (C) SNU387 and (D) SNU398 liver cancer cells. FLLL32 also inhibited the expression of Cyclin D1 and/or Bcl-2, DNMT1 and increased the cleavages of caspase-3 and PARP. [file 1476-4598-9-217-S1.PPT]

## Slide 1
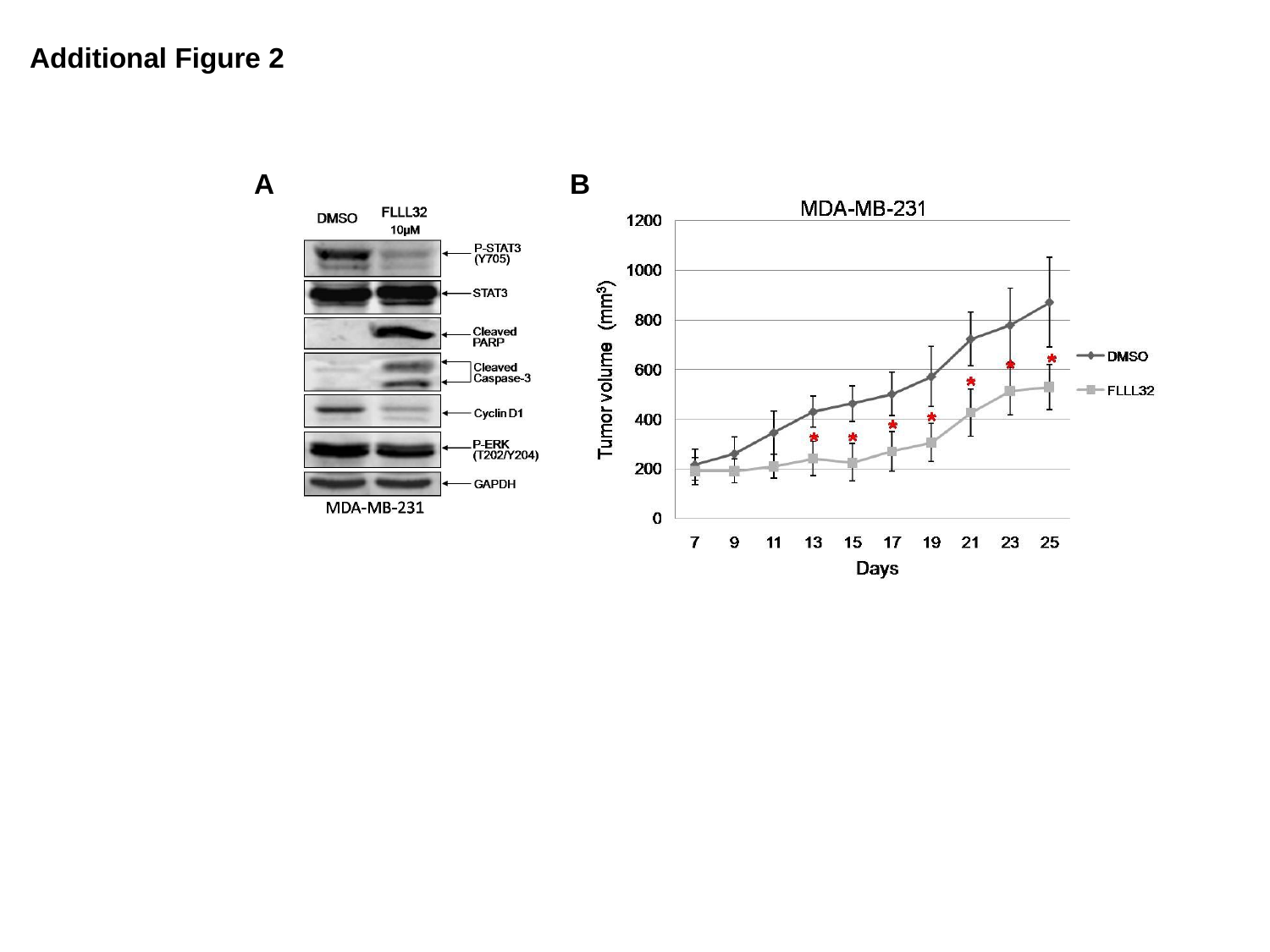

Additional Figure 2
A B

Supplement: Additional file 2 — The effect of FLLL32 on tumor growth in vivo. (A) FLLL32 inhibited STAT3 phosphorylation and induced apoptosis in MDA-MB-231 breast cancer cells lines; (B) FLLL32 suppressed the growth of MDA-MB-231 xenograft tumors in NOD/SCID mice. [file 1476-4598-9-217-S2.PPT]
